# Mutant torsinA in the heterozygous DYT1 state compromises HSV propagation in infected neurons and fibroblasts

**DOI:** 10.1038/s41598-018-19865-2

**Published:** 2018-02-02

**Authors:** Bence György, Lilian Cruz, David Yellen, Massimo Aufiero, Isabel Alland, Xuan Zhang, Maria Ericsson, Cornel Fraefel, Yu-Ching Li, Shuko Takeda, Bradley T. Hyman, Xandra O. Breakefield

**Affiliations:** 1 Molecular Neurogenetics Unit, Department of Neurology and Center for Molecular Imaging Research, Department of Radiology, Massachusetts General Hospital and Program in Neuroscience, Harvard Medical School, Boston, MA 02114 USA; 2 Department of Cell Biology, Harvard Medical School, Boston, MA 02115 USA; 3 Institute of Virology, University of Zurich, Zurich, 8057 Switzerland; 4 Department of Neurology, College of Medicine, University of Florida, Gainesville, FL 32610 USA; 5 Department of Neurology, Massachusetts General Hospital, Massachusetts General Institute of Neurodegenerative Diseases (MIND), Charlestown, MA 02129 USA; 6 Massachusetts Alzheimer Disease Research Center, Charlestown, MA 02129 USA; 7 Department of Neurobiology, Harvard Medical School, Boston, MA 02115 USA

## Abstract

Most cases of early onset torsion dystonia (DYT1) are caused by a 3-base pair deletion in one allele of the *TOR1A* gene causing loss of a glutamate in torsinA, a luminal protein in the nuclear envelope. This dominantly inherited neurologic disease has reduced penetrance and no other medical manifestations. It has been challenging to understand the neuronal abnormalities as cells and mouse models which are heterozygous (Het) for the mutant allele are quite similar to wild-type (WT) controls. Here we found that patient fibroblasts and mouse neurons Het for this mutation showed significant differences from WT cells in several parameters revealed by infection with herpes simplex virus type 1 (HSV) which replicates in the nucleus and egresses out through the nuclear envelope. Using a red fluorescent protein capsid to monitor HSV infection, patient fibroblasts showed decreased viral plaque formation as compared to controls. Mouse Het neurons had a decrease in cytoplasmic, but not nuclear HSV fluorescence, and reduced numbers of capsids entering axons as compared to infected WT neurons. These findings point to altered dynamics of the nuclear envelope in cells with the patient genotype, which can provide assays to screen for therapeutic agents that can normalize these cells.

## Introduction

Early onset torsion dystonia (DYT1) is a dominantly inherited neurologic disease causing muscle contractions and abnormal movements, with no other symptoms^1^. Most cases are caused by a three base pair deletion in one allele of *TOR1A* resulting in loss of a glutamic acid residue in the carboxyl terminal region of torsinA, a protein located in the contiguous lumen of the nuclear envelope (NE) and endoplasmic reticulum (ER)^2,3^. TorsinA is a member of a family of proteins termed ATPases associated with various cellular activities (AAA+)^2,4^ and forms a hexameric ring structure with one of two other transmembrane proteins, LULL1 or lamina associated polypeptide 1 (LAP1)^5,6^. Affected individuals are heterozygous (Het) for wild-type (WT) and mutant torsinA alleles and the disease phenotype has low penetrance (only 30–40% of mutant gene carriers are affected)^1^. Current therapies for DYT1 dystonia include anticholinergic drugs^7^, deep brain stimulation^8^ and local injections of botulinum toxin^9^, all of which can have complications and/or are only partially effective.

In order to develop new therapies for dystonia it is important to generate assays to screen for drugs or genes that can normalize DYT1 genotypic cells. Several potential assays are available, including aggregates formation by overexpressed mutant torsinA^10,11^, decreased ability to release *Gaussia* luciferase through the secretory pathway^12^, and increased sensitivity to ER stress^13,14^ in cells expressing the mutant allele as compared to controls. When assessed in skin fibroblasts, however, these assays might be confounded by variations in fibroblast subtypes and passage number. Based on studies indicating that torsinA is involved in replication of Herpes Simplex Virus type 1 (HSV)^15–18^, we sought to develop a more robust assay to evaluate normalization of function in genotypic DYT1 cells.

HSV DNA enters the nucleus through the nuclear pores^19^ and replicates in the nucleus where its genome is packaged into capsids (for review see^20^). These capsids then exit the nucleus by budding out from the inner nuclear membrane (INM) and forming transitory enveloped intermediates in the lumen of the NE which then fuse with the outer nuclear membrane (ONM) releasing the capsids into the cytoplasm. The capsids then acquire the final envelope during exit from the cells (for a review see^21^). TorsinA has been implicated in NE topography by its association with LAP1^22^ and SUN proteins^23,24^, which span the INM, and with nesprins^25^ and LULL1^24^, which span the ONM. Torsin in *Drosophila* is critical in release of large ribonuclear protein particles from the nucleus into the cytoplasm by a similar NE budding mechanism^26,27^. TorsinA is also associated with chaperone proteins in the ER involved in protein processing through the secretory pathway (for review see^28^).

In this study, we took advantage of a replication competent variant of HSV in which a capsid protein, VP26, is fused to monomeric red fluorescent protein (RFP-VP26)^29^. This variant HSV was used to monitor plaque number and size in human DYT1 and control fibroblasts. In addition, we monitored viral replication by fluorescent and electron microscopy in nuclei and cytoplasm of neurons cultured from mouse embryos – WT, Het or homozygous for knock-in (KI) of the DYT1 mutation in the *Tor1a* gene^30^. We also tracked the movement of labeled capsids down axons in these neurons using microfluidic chambers. We found a decrease in viral plaque number and size in DYT1 compared to control fibroblasts, and decreased replication of HSV in neurons homozygous for the DYT1 mutation (KI) compared to Het or WT. Both Het and KI neurons showed a decrease in nuclear egress of the HSV capsids into the cytoplasm, as compared to WT neurons. We also observed higher frequency of blebbing of the NE in uninfected and infected KI neurons. Thus, HSV provides a probe to distinguish the WT and Het genotypes, with the latter being genotypically similar to DYT1 patients, with significant parameters including reduced propagation in patient fibroblasts, and reduced viral fluorescence in the cell body in Het, as compared to WT neurons.

## Results

### HSV replication in DYT1 and control fibroblasts

**Figure 1:**
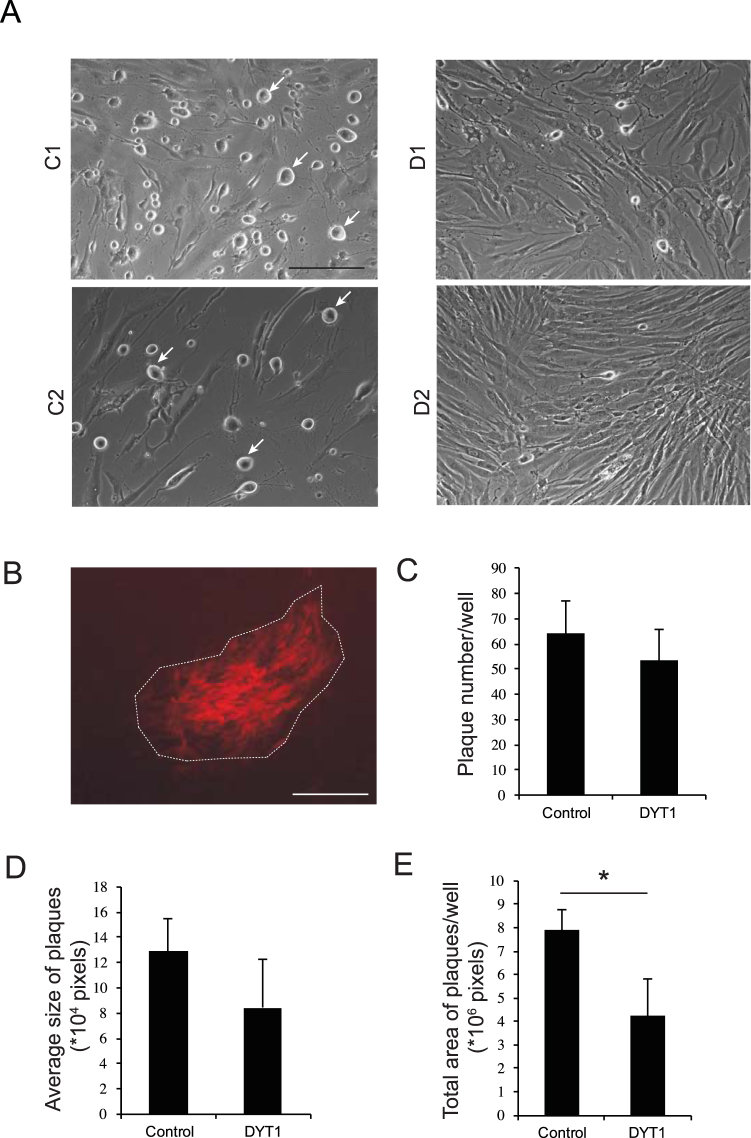
HSV replication dynamics in DYT1 and control fibroblasts. Human patient and control fibroblasts were infected with HSV-VP26-RFP and cytopathic effect and plaque formation were evaluated. (**A**) Cytopathic effect of the virus (M.O.I. 0.001) was more pronounced in two control lines (GM05659, C1; GM08424, C2) (rounded cells) than in two DYT1 patient derived lines (MIN30857, D1; D2306, D2) at 44 h post-infection (200x magnification, scale bar is 50 μm). (**B**) An example of a red fluorescent viral plaque in GM08424 cells in the semisolid 0.4% agarose medium (40x magnification, scale bar is 250 μm). The plaque number (**C**), plaque size (**D**) and total plaque area (**E**) was determined using ImageJ in DYT1 lines (n = 3, MIN30857 GM02306 and GM02551) and control lines (n = 3, GM05659, GM08424, NHF74). Experiments were always performed in pairs with one DYT1 and one control line. On average DYT1 cultures had significantly decreased total plaque area (plaque number x plaque size) as compared to control cultures (p < 0.033, paired t test).

Based on the findings of others that cells with alterations in torsinA expression showed decreased ability of HSV to replicate, and specifically to exit the NE^15–18^, we evaluated this for the first time in skin fibroblast cultures derived from DYT1 patients and controls. Confluent cultures were infected at a multiplicity of infection (M.O.I.) of 0.001 virions per cell and evaluated microscopically post-infection at different time points. Based on cytopathic effect (rounded, floating cells) the DYT1 lines appeared more resistant to HSV replication and lysis, shown here at 44 h post-infection (Fig. 1A). Based on this marked difference in cytopathic effect, we aimed at developing a more quantitative assay. Thus, we performed a fibroblast HSV plaque assay, in which virus was restrained from extracellular diffusion in semi-solid medium and thus propagated by infecting only neighboring cells. The number and size of the plaques are indicative of the efficiency of virus replication. We evaluated the number and size of fluorescent plaques (Fig. 1B) using different M.O.I.s of HSV-VP26-RFP, ranging from 10^−1^ to 10^−4^. There was a trend for a decrease in both the number (Fig. 1C) and size of plaques (Fig. 1D) in DYT1 lines (n = 3), as compared to control lines (n = 3). When the total plaque area was determined (number of plaques x area of individual plaques) the area was significantly decreased in DYT1 as compared to control lines, supporting a reduced rate of replication and/or cell-to-cell spread in patient cells (Fig. 1E).

### Replication of HSV in neurons cultured from mouse embryos

**Figure 2:**
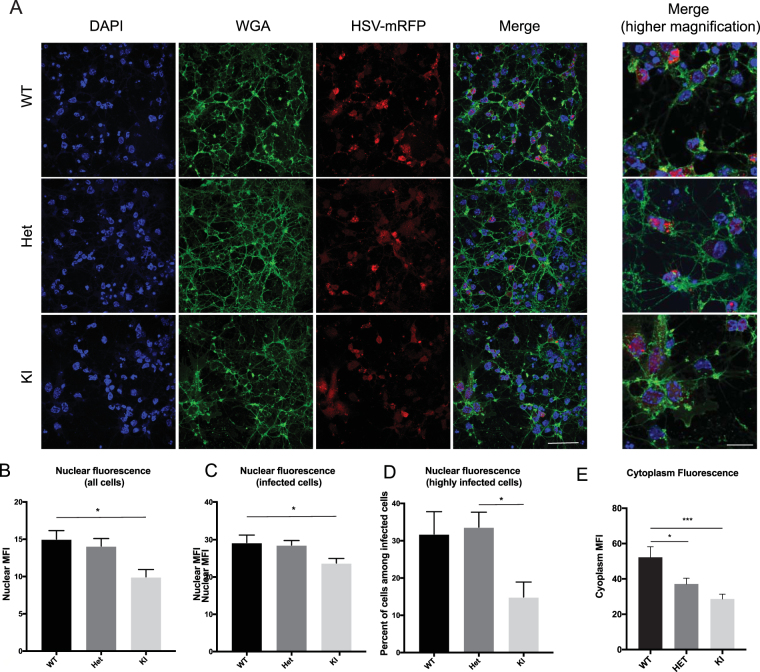
Monitoring HSV infection in nucleus and cytoplasm of neurons. (**A**) Representative images of WT, Het and KI lines infected with HSV-VP26-RFP at the M.O.I. of 1 at 8 h post-infection. Cells were stained for DAPI (blue), WGA-488 (green) and virus is shown in red. Scale bar is 50 μm, for higher magnification images is 20 μm. (**B**–**D**) Mean fluorescent intensity (MFI) in the red channel inside the nucleus for all cells, approximately 600 cells were analyzed per genotype (**B**), or only infected cells (having a mean fluorescent intensity two-times above background, **C**). We also analyzed the percentage of highly infected (three-times above background level) cells among all infected cells (**D**). ANOVA was used to compare groups followed by Tukey’s post-hoc test for pairwise comparisons, *p < 0.05. (**E**) Cytoplasm fluorescence was measured by ImageJ for all cells. ANOVA was used to compare groups followed by Tukey’s post-hoc test for pairwise comparisons. WT samples had a significantly higher HSV concentration in the cytoplasm than Het (*p < 0.05) and KI (***p < 0.001) samples. Approximately 500 cells were analyzed from each genotype.

Since DYT1 is a neurologic disease, we also evaluated virus replication of HSV-VP26-RFP in neuronal cultures prepared from WT and torsinA mutant Het and homozygous KI embryos. Cultures were generated from matings of Het mice carrying a KI allele of the GAG deletion in the *Tor1a* gene^30^. Neuronal cultures were established from individual E15 embryos, at which time embryos are still viable. (*Note*. Homozygous KI mice die at birth.) Neurons were infected with HSV-VP26-RFP at an M.O.I. of 1. Image analysis at 8 h post-infection was used to determine the relative level of RFP fluorescence in the nucleus and cytoplasm using DAPI to stain nuclei and WGA (wheat germ agglutinin)-488 to determine the outline of cell bodies (Fig. 2A). Considering all neurons (Fig. 2B) or only infected neurons (Fig. 2C) (600 cells of each genotype were analyzed for (B) and (C)), there was a significant decrease in the level of RFP fluorescence in the nuclei of KI neurons compared to WT and Het neurons. We also analyzed the percentage of highly infected cells among all infected cells and found that there were significantly fewer highly infected cells in the KI genotype compared to Het and WT cultures (Fig. 2D). In the same cell populations (500 cells of each genotype) there was markedly less RFP fluorescence in the KI cell bodies compared to the WT and Het neurons, also with a significant reduction in the Het compared to the WT neurons (Fig. 2E). This is consistent with a decreased level of viral replication in KI, as compared to Het and WT neurons, with comparable levels of viral replication in WT and Het neurons, and a decrease in nuclear egress of virions in both Het and KI cells, as compared to WT.

**Figure 3:**
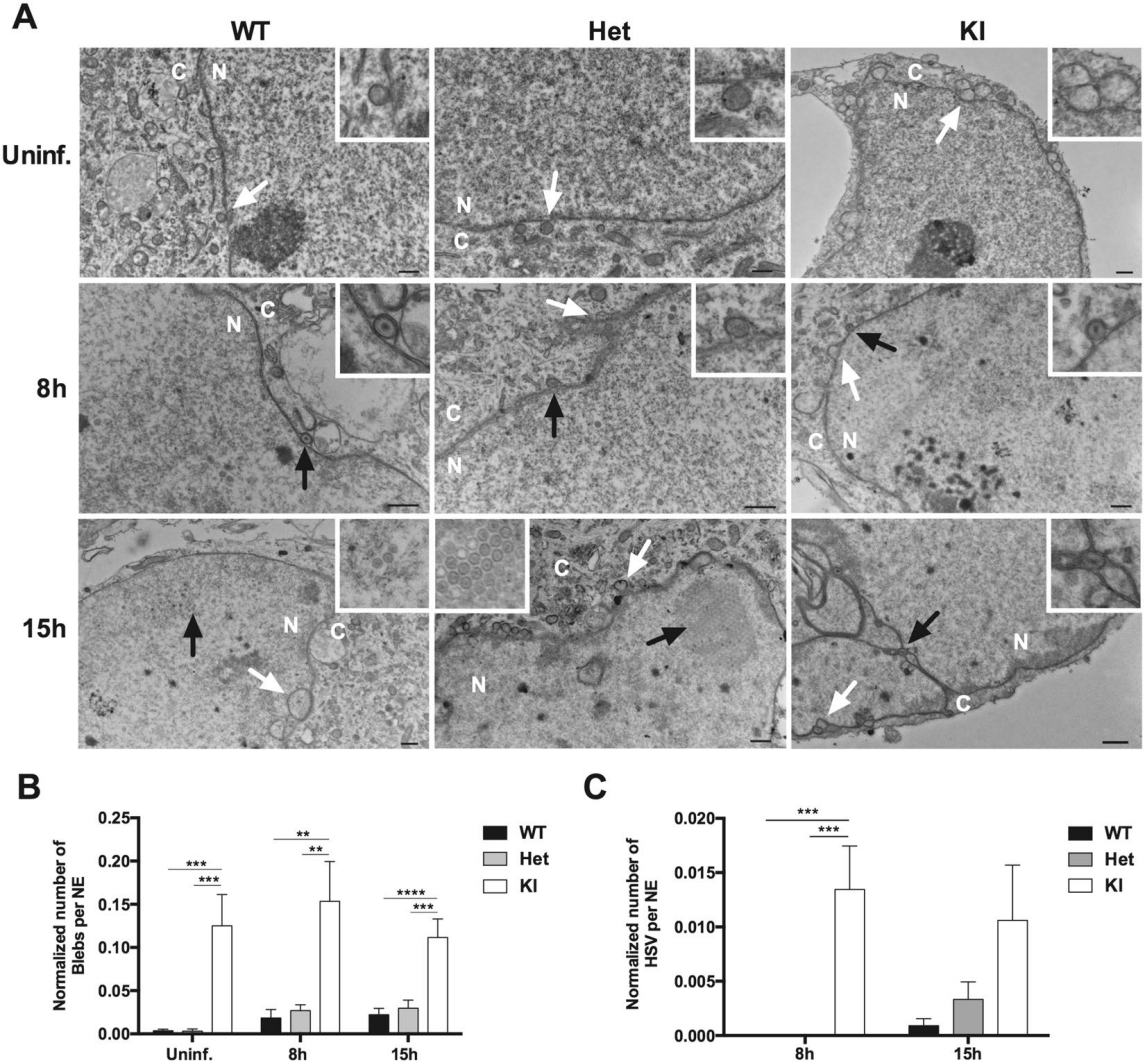
HSV infection and bleb formation in DYT1 neurons. (**A**) WT, Het, and KI neurons were uninfected (Uninf.) or infected with HSV-VP26-RFP (M.O.I. of 1) and imaged using an electron microscope at 8 h and 15 h post-infection. White arrows indicate blebs associated with the NE. Black arrows indicate HSV particles either associated with the NE (8 h and 15 h) or in the nucleus (15 h). Enlarged images in the top right corners represent the area indicated by white arrows (uninfected) or black arrows (infected). (**B**) At all three conditions (uninfected, 8 h and 15 h post-infection), KI neurons had significantly more blebs associated with the NE than WT (uninfected: p = 0.0002, n = 50; 8 h: p = 0.0022, n = 30; 15 h: p < 0.0001, n = 30) or Het cells (uninfected: p = 0.0002, n = 50; 8 h: p < 0.0045, n = 30; 15 h: p = 0.0003, n = 30). (**C**) HSV particles associated with the NE were counted from a total of 30 neurons for each condition. At 8 h post-infection, a significant difference was seen in the average number of HSV particles associated with the NE in KI neurons, as compared to WT (p = 0.0003) and Het (p = 0.0003). There was no significant difference among any of the samples at 15 h post-infection. (**B** and **C**) Number of blebs and HSV in NE was normalized by the length (perimeter) of the NE. Statistical significance was determined using one-way ANOVA followed by a post-hoc Tukey test. N: nucleus; C: cytoplasm. Scale bars: 500 nm.

Given the localization of torsinA in the contiguous lumen of the NE and ER and the budding of HSV capsids out of the nucleus through the NE, we carried out electron microscopic evaluation of the NE in mouse neurons cultured from WT, Het and KI embryos. Neurons were non-infected or infected with HSV-VP26-RFP at M.O.I. of 1. Samples were analyzed at 8 and 15 h post-infection (Fig. 3A). The uninfected WT and Het neurons showed a generally intact NE with occasional associated vesicular structures, while many of the KI neurons showed marked vesiculation of the NE, termed here blebs (Fig. 3B, Supplementary Table 1, p < 0.0001, chi-square test, between genotypes). This NE vesiculation remained high in the KI neurons at 8 h and 15 h post-infection (p = 0.008 and p = 0.001, respectively, chi-square test, between genotypes). Representative images of the cells at 8 h post-infection revealed a few fully formed capsids associated with the NE in WT and Het cells, while in KI cells there was a marked increase in NE-associated capsids (Fig. 3C, Supplementary Table 1, p = 0.009 for 8 h, chi-square test, between genotypes). After 15 h post-infection, there was a trend for more capsids associated with the NE for Het as compared to WT cells, but this was not significant (Fig. 3C). At 15 h post-infection viral replication centers within the nucleus were apparent for all genotypes (Supplementary Fig. 1, Supplementary Table 1, not significant between genotypes, chi-square test).

**Figure 4:**
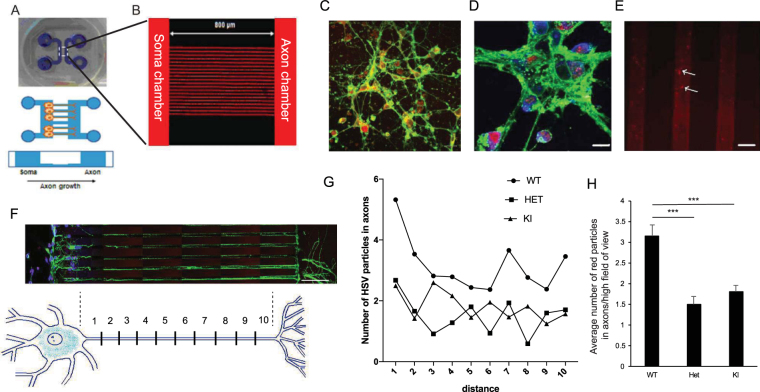
Movement of HSV-VP26-RFP capsids down neuronal axons. Neurons from E15 mouse embryos were plated in microfluidic chambers. (**A** and **B**) Layout of microfluidic device. (**C** and **D**) Five days later WT neurons were fixed and stained for tau (green) and DAPI (blue). The virus is shown in the red channel. (**E**) Neurons were infected with HSV-VP26-RFP (M.O.I. of 10) and HSV capsids (red; arrows) were visualized within microgrooves at 8 h post-infection. Scale bars: 30 μm (**C**), 10 μm (**D**,**E**). (**F**) Microscopic image and schematic of the analysis with the axon divided into zones 1 to 10 (each zone length is around ~80 μm, scale bar is 100 μm). Microscopic image is a tile scan of 14 images (10 were captured in the microgrooves, two in the cytoplasm and two at the end of the processes). Zone 1 represents the proximal part, while zone 10 is the most distal part of the axon still detectable in the microgroove. (**G**) Number of virus particles in different axonal zones. No significant trend was found for any genotype (linear regression analysis, R^2^ is 0.21, 0.08 and 0.34 for WT, Het and KI, respectively, not significantly different using F-test (p = 0.18, p = 0.40, p = 0.07 for WT, Het and KI, respectively)). (**H**) Overall, throughout the axons (average of zones 1 to 10) there were significantly higher numbers of virus capsids in the WT neurons than either the Het or KI neurons, (p < 0.001, ANOVA followed by Tukey’s post hoc test) (WT: n = 6 embryos, Het: n = 6 embryos, KI: n = 5 embryos; 6 axons per embryo were analyzed and 10 images captured per axon, in 3 separate experiments).

To obtain a clearer picture of where the “hang up” is in HSV propagation in neurons with mutant torsinA, neurons were plated in microfluidic chambers which allow them to extend axons into microgrooves, while retaining cell bodies and dendrites in the plating chamber (Fig. 4A and B)^31^. Cultures were verified to be >90% neurons by staining for beta-III tubulin (data not shown). Five days after plating neurons, dendritic processes were visible in the plating chamber. Then neuronal cell bodies were infected with HSV-VP26-RFP (at  M.O.I. of 10) and 8 h later virus could be visualized by fluorescent microscopy in the cell bodies (Fig. 4C and D) and in axons that had extended into the microgrooves (Fig. 4E,F). The axon chambers were divided into zones by distance along the microgroove (Fig. 4F) and the number of capsids in each zone was imaged and counted for WT, Het and KI neurons and averaged over 3 separate experiments (Fig. 4G). We did not see a decrease in the rate of movement of HSV particles down the axons from zone 1 to 10 (linear regression and F-test, not-significant for any genotype). The average number of capsids in the axons was about twice as high in WT compared to Het and KI neurons (Fig. 4H). This data suggests that there is no deficiency of the transport of HSV particles down the axons and that lower overall number of HSV particles in the axons probably reflects decreased replication and/or nuclear egress of HSV from the nucleus in Het and KI cells.

## Discussion

Our studies are unique in that we evaluated HSV propagation in human DYT1 patient and control fibroblasts, as well as in mouse embryonic neurons bearing the same mutation in torsinA in the Het state (genotypically similar to patients) and in the homozygous state, as well as in WT neurons from littermates. We are the first to focus on cells bearing the GAG deletion in the Het state in both human *TOR1A* and mouse *Tor1a* genes. In order to facilitate visual monitoring of virus infection, we used a HSV variant in which one of the capsid proteins, VP26 is fused to RFP^29^. We found that HSV replication is compromised in these Het genotypic DYT1 cells. In the case of fibroblasts, plaque size and number were reduced in patient as compared to control cells, which is consistent with reduced viral yield and/or restricted exit from the nucleus or cell. In neurons Het for the mutation, and even more so in neurons homozygous for it (KI), this defect in HSV replication appears to be related to a compromise of function in the NE resulting in an apparent delay in egress of newly replicated capsids from the nucleus through the NE to the cytoplasm. This NE compromise in neurons may relate to their high dependence on torsinA, rather than torsinB, as torsinB can compensate for defects in torsinA^32^. Here we provide culture assays to evaluate compromise of torsinA function in genotypic DYT1 cells, including plaque formation in DYT1 fibroblasts, and relative number of HSV capsids in cell bodies and axons of infected neurons. Both assays provide a means to screen for therapeutic agents that can restore normal torsinA function. In addition, the abnormality in Het neurons supports an inherent compromise of NE integrity in neurons caused by the expression of mutant torsinA.

Our studies confirm findings by others that the status of torsinA in cells can affect replication of HSV. Overexpression of WT torsinA in pheochromocytoma cells was associated with blebbing of the NE and reduced virus yields^15^. This is consistent with a number of other studies that have documented aberrations in the structure of the NE in different cell types when torsinA is overexpressed^3,10,33–35^. In mouse embryonic fibroblasts (MEFs) null for torsinA, HSV infection yielded fewer virus particles than in WT MEFs with vesiculation of the NE in the former, which increased with viral infection^16^. In yet another cell type, HeLa cells, knock-out of both torsinA and torsinB caused only a marginal reduction in viral production, while knock-out of their binding partner, LULL1 caused a marked decrease^17^.

TorsinA is AAA+ protein located in the continuous lumen of the NE and ER^28^, with other members of this family acting as molecular machines which generate mechanical force^36^. Together with associated proteins torsinA has a role in dynamic processes in both the NE and ER involved in structure and function of these compartments. Converging lines of evidence implicate torsinA, together with its binding partners, LAP1 and LULL1, in morphologic and topographic changes in the NE related to nuclear orientation in cell migration, placement of nuclear pore complexes and egress of large particles out of the nucleus into the cytoplasm. Early findings demonstrated LULL1 and LAP1 in the NE as binding partners for torsinA^4,37–39^, as well as the appearance of interluminal vesicles in the NE of neurons in homozygous torsinA mutant mouse embryonic brain^37^. These vesicles (also call buds or blebs) were found only in neurons during development, but were recapitulated in embryonic stem cells from mice bearing homozygous KI of the GAG mutation differentiated into neurons in culture^40^. Subsequent studies have shown that LULL1 and its homologue LAP1 form a heterohexamer with torsinA, which has higher ATPase activity with WT than with mutant torsinA^5,41^. TorsinA also associates with nesprins, which link the ONM of the NE to the cytoskeleton^25^, and with SUNs, which link the INM to nuclear filaments^24^, and as such has the potential to regulate the linker of nucleoskeleton and cytoskeleton (LINC) complex^42^. Recent studies indicate that torsinA is also involved in the fission of vesicles, containing large ribonuclear protein particles, which bud from the INM into the lumen of the NE (similar to HSV), such that interference with torsinA function causes these budding vesicles to be “stuck” to the INM^27^. Components of this budding process are shared with nuclear pore formation, which is also dependent on torsinA function^39^.

In addition to its role in NE morphology and dynamics, torsinA has been implicated in ER functions, including processing proteins through the secretory pathway, with mutant torsinA predisposing cells to ER stress^13,14,43,44^. Assays have been developed around these functions, as well as the tendency of overexpressed mutant torsinA to form whorled membrane inclusions^3^, in order to screen for drugs that might normalize cellular dysfunction caused by the presence of mutant torsinA^45^. The HSV assays developed here have the advantage that they rely on endogenous levels of torsinA and can be carried out in patient fibroblasts, as well as in embryonic mouse neurons that have the DYT1 patient genotype. Since HSV-VP26-RFP is fluorescent, there is no need for other staining to monitor the propagation of this virus. Three assays are described here: one monitoring viral plaque size and number in monolayer cultures, one monitoring viral replication in the nucleus and egress into the cytoplasm by virus fluorescence levels, and one quantitating virus entry into neuronal axons in microfluidic chambers, all of which gave significant differences between the DYT1 and WT genotypes. It seems feasible that these assays could be developed into a moderate high throughput screen using computerized visual monitoring of fluorescent capsids.

As evaluated by levels of HSV fluorescence in the cell nuclei, there was decreased replication of the virus in the nuclei of KI cells, as compared with Het and WT cells. We hypothesize two possible ways that the TOR1A mutation might suppress HSV replication in the nucleus. First, during initial infection of a cell the viral DNA is threaded into the nucleus through the nuclear pores in the NE^19^. Others have found that loss of function of torsin proteins can perturb formation and location of nuclear pores^39,46^. Therefore, mutant torsinA may lead to a delay or inefficiency in the viral DNA entering the nucleus, which would in turn result in reduced replication in the nucleus. Second, it is known that during viral infection, viruses hijack the host translation apparatus to produce large amounts of viral proteins, which causes ER stress. To try to restore ER homeostasis, cells initiate the unfolded protein response (UPR) to alleviate the effects of ER stress. The IRE1/XBP1 pathway is the most conserved UPR branch, and it activates ER-associated protein degradation (ERAD) to reduce the ER load. During HSV infection the viral UL41 suppresses the IRE1/XBP1 signal pathway by decreasing expression and splicing of XBP1 mRNA^47^. Because expression of mutant torsinA elevates ER stress and UPR signal pathways^48,49^, it may take higher levels of the UL41 viral protein to suppress ER stress so that viral proteins important in viral DNA replication can be translated efficiently.

The high frequency of the glutamic acid deletion in torsinA in cases of DYT1 in the Ashkenazi Jewish population can be explained by a founder mutation following pogroms in Lituania/Byelorussia 20–30 generations ago^50^. It is intriguing to consider that there might be a relationship between the DYT1 mutation and viral selection. In one scenario, the decreased ability of DYT1 individuals to propagate viruses that have to pass out of the nucleus as part of their replicative cycle might provide some selective advantage in protecting individuals from viral infections. In another scenario, subtle abnormalities in the NE in DYT1 neurons may make them more susceptible to debilitating conditions, such as viral infection, which could be related to penetrance and manifestation of the mutation as a movement disorder. Further work will be needed, for example, to see if DYT1 carriers have a decreased rate of HSV infection compared to non-carriers, or whether viral infection is associated with onset of symptoms.

Herein, we describe several assays for evaluating the effects of mutant torsinA on propagation of HSV that can be applied to visual moderate-high throughput screens for drug candidates for therapy. The finding that neurons with one WT allele and one mutant allele, or two mutant alleles, showed a retardation of HSV release from the nucleus, supports a dominant-negative action of this mutant protein in the presence of WT torsinA. In addition to drug screens, these assays can be applied to evaluating the corrective effects of gene therapy – by delivering more WT torsinA to cells via a viral vector, or using CRISPR technology to disrupt or correct the mutant allele in the Het state as a first step toward preclinical efficacy studies.

## Conclusion

Assays are described to monitor the effect of mutant torsinA at endogenous levels on the propagation of HSV infection by plaque size and number in human fibroblasts, and by levels of viral fluorescence in genotypically DYT1 mutant neurons in microfluidic chambers as a critical step in evaluating drugs and gene therapy protocols aimed to normalize this phenotype and thus serve as candidates for therapeutic intervention.

## Materials and Methods

### Cell culture and HSV stocks

Experimental protocols involving human cells were approved by the Massachusetts General Hospital Institutional Review Board (IRB, approval number: 2009p-001500) and all experiments were performed in accordance with the guidelines in the approved IRB protocol. Primary fibroblast lines including from controls: NHF74, MIN33113, GM5659, GM08424, and DYT1 patients: GM02551, MIN30857, GM2306 were cultured in Dulbecco’s Modified Eagle Media (DMEM; Gibco, Rockville, MD USA) supplemented with 10% fetal bovine serum (FBS) and 1% penicillin/streptomycin (Gibco). GM lines were obtained from the Coriell Institute, NHF from the Breakefield laboratory, and MIN from Mass. General Hospital, all obtained and used under the relevant IRB guidelines. Informed consent was obtained from all subjects. Cells were used for experiments under passage number 15. Cultures were negative for mycoplasma contamination, as assessed using the MycoAlert Mycoplasma Detection Kit (Lonza, Walkersville, MD USA). Sanger sequencing (see below) was used to verify the presence or absence of the DYT1 mutation.

Replication competent, attenuated HSV tagged by fusion of RFP with the VP26 capsid protein (HSV-VP26-RFP) was propagated in Vero (African green monkey, kidney epithelial, ATCC-CCL-81) cells^29^. Cells were maintained in DMEM with 10% FBS and 1% penicillin/streptomycin. HSV stocks were generated, as described^51^.

### Animals

DYT1 Het and KI mice^30^ were bred and housed in our facility. Experiments were approved by the Institutional Animal Care and Use Committee at Massachusetts General Hospital, Boston. All animal procedures were performed in accordance with the protocol approved by the Institutional Animal Care and Use Committees at the Massachusetts General Hospital (Boston, MA, USA (protocol number: MGH/2017N000047)).

### HSV replication assay in fibroblasts

Human DYT1 patient-derived and control fibroblasts were infected with HSV-VP26-RFP under different conditions. For the virus-induced cytopathic effect experiments, we used a range of M.O.I. Cells were viewed under the microscope at 12, 20, 24 and 44 h post-infection. For the plaque assay, we plated 100,000 cells/well in a 12-well plate. Two days later, when the cells reached confluency, we added HSV-VP26-RFP in serial dilutions, ranging from 10^−5^ to 1 plaque forming units (PFUs) for 6 h at 37 °C. Then we aspirated the virus-containing culture media and overlaid 0.4% agarose dissolved in media + serum. The plates were left at room temperature for 30 min to allow for solidification of the agarose. Forty-eight to 72 h later, we visualized the red plaques using epifluorescent microscopy. Finally, we counted the number and measured the area of red plaques in each well using ImageJ v1.43.

### Fluorescent evaluation of HSV-VP26-RFP replication in neurons

Timed pregnancies of DYT1 Het mice^30^ were monitored in-house and dissociated cortical neuronal cultures were prepared from individual E15 embryos, as described^52^. Cells from each embryo were saved for genotyping using primers described^30^. Mouse cortical neurons from WT, Het and KI embryos were plated on coverslips pre-coated with poly-D-lysine and laminin (Corning, New York, NY USA). The purity of neuronal cultures was determined by beta-III tubulin staining (data not shown).

Neurons were infected in triplicate with HSV-VP26-RFP at the M.O.I. of 1. Cells were analyzed uninfected and 8 h and 15 h post-infection. Cells were fixed for 20 min with 4% paraformaldehyde (PFA) in phosphate buffered saline (PBS) and then stained for DAPI (1:10,000; Life Technologies) and WGA-488 (10 µg/mL; Invitrogen, Carlsbad, CA USA). Images were captured with a Zeiss LSM 710 inverted confocal microscope (Carl Zeiss AG, Germany) using a 63x oil objective (plan-apochromat SF25, DIC, NA = 1.4) and a *z* thickness of 1 μm. Eighteen images were taken for each embryo. Cytoplasm fluorescence was analyzed by ImageJ version 2.0.0-rc-54/1.51 h (Supplementary Fig. 2). Briefly, images were acquired in three channels (red, blue, and green) and overlaid into composites. The images were analyzed using ImageJ v2.0.0 to quantify the red fluorescence signal, indicating the intensities of HSV-VP26-RFP, in the cytoplasm of cells from each genotype (WT, Het and KI). To select only nuclear areas in the images, the threshold tool was used in ImageJ for the DAPI channel, and then the red fluorescence intensity within the selected regions was measured using the Analyze Particles tool again with an optical thickness of 1 μm. This value was subtracted from the total red fluorescence intensity for the image to determine the fluorescence value for the cytoplasm^53^. For nuclear fluorescence analysis, we segmented cells based on DAPI staining using Imaris 9.0.0 (Bitplane, Zurich, Switzerland) (Supplementary Fig. 3). RFP fluorescence intensity was determined for all cells or only for infected cells. When analyzing all cells, no thresholding was done, i.e. infected and also non-infected cells (having background red fluorescence) were included in the analysis (data is presented in Fig. 2B). We also analyzed nuclear fluorescent intensity only in infected cells (Fig. 2C). In this case, cells that had a nuclear fluorescent intensity twice as high as the background level were considered ‘infected’. Finally, we analyzed the percentage of highly infected cells (defined as having 3 times above the background level) among all infected cells (Fig. 2D). Image analysis was performed in a blinded manner with the genotypes unknown to the person performing the analysis.

### Electron microscopy

Mouse cortical neurons from WT, Het and KI embryos were cultured on coverslips pre-coated with poly-D-lysine and laminin (Corning). Cells were infected with HSV-VP26-RFP at an M.O.I. of 1 PFU/cell and fixed 8 and 15 h after infection. The fixative (5% glutaraldehyde 2.5% PFA, 0.06% picric acid in 0.2 M sodium cacodylate buffer (pH 7.4)) was diluted 1:1 with culture media in the dish; the cells (on coverslips) were fixed for 1 h, washed 3x in 0.1 M cacodylate buffer, then postfixed in 1% osmium tetroxide (OsO4)/1.5% potassium ferrocyanide (KFeCN6) for 30 min, washed in water 3x and incubated in 1% aqueous uranyl acetate for 30 min followed by 2 washes in water and subsequent dehydration in grades of alcohol (5 min each; 50%, 70%, 95%, 2 × 100%). Cells were embedded in plastic by inverting a gelatin capsule filled with Epon/Araldite on top of the coverslip and polymerizing at 60 °C for 24 h. After polymerization, the coverslip was removed by dipping the block in LN2. Ultrathin sections (about 80 nm) were cut on a Reichert Ultracut-S microtome, picked up on to copper grids, stained with lead citrate and examined in a JEOL 1200EX. Transmission electron microscope and images were recorded with an AMT 2k CCD camera at the Harvard Medical School Electron Microscopy Facility. Number of cells containing blebs (vesicular structures associated with the NE) and the number of blebs associated with the NE were counted in uninfected (n = 50 cells) and HSV infected (8 h and 15 h) samples (n = 30 cells of each genotype) in a blinded manner. The number of cells containing HSV replication centers and HSV particles associated with the NE were quantified in infected samples (n = 30 cells of each genotype).

To determine if WT, Het, and homozygous KI neurons had qualitative differences in nuclear morphology when infected with HSV, a blinded test was conducted in which unlabeled EM images were classified as WT, Het, or homozygous KI based on a given set of characteristics that most closely represent each genotype. The criteria used were as follows: WT cells should have intact NEs with distinct inner and outer membranes without bulges between them, and a relatively high number of virus particles in the nucleus and cytoplasm; both Het and KI cells should have virus particles “stuck” within the NE lumen or associated with the ONM; KI cells should have irregular shaped vesicles, or “blebs,” between the INM and ONM. Nineteen WT, 24 Het, and 52 KI images were evaluated. Overall, the genotypes of the images were identified with 66% accuracy. WT and Het cells were distinguished from each other with 81% accuracy, WT and KI with 90% accuracy, and Het and KI with 78% accuracy.

### HSV-VP26-RFP axonal transport assay

Mouse cortical neurons from WT, Het and KI embryos (see above) were plated at a density of 1.5 × 10^4^ cells/chamber in poly-D-lysine coated 2-chamber microfluidic devices designed to separate cell soma and dendrites from axons^31^, which were generated in the MGH BioMEMs facility, in Neurobasal medium + 1x B27 (Thermo Fisher Scientific, Lafayette, CO USA) + 1% penicillin/streptomycin (Corning). Chambers consisted of a molded elastomeric polymeric piece placed against a glass coverslip which can be used for immunocytochemistry and real-time imaging, as well as isolation of cell compartments^31,54^. HSV-VP26-RFP was added to the soma chamber at an M.O.I. 10 PFU/cell. Cells were cultured for 8, 15 and 24 h before fixing with 4% PFA in PBS. PFA was exchanged to PBS after 30 min and cells were stained for tau (monoclonal antibody DA9, 1:10,000, courtesy of Dr. Peter Davies)^55^ and detected with Alexa fluor 488 anti-mouse secondary antibody (Invitrogen). The nuclei were stained with TO-PRO3 (Thermo Scientific, 1:1000) for 5 min at room temperature. The axonal presence of HSV-VP26-RFP capsids was analyzed with a Zeiss LSM 710 confocal microscope using a 40x oil objective (EC Plan-Neofluor, DIC, NA = 1.3). The microscope was programmed to perform a tile scan starting from the soma down to the distal end of the axonal process using autofocus on the tau staining (Alexa-488 channel) in each frame. The experiment was repeated on 3 different occasions. Image analysis was done in ImageJ. Briefly, we divided the axon length into 10 equal zones, with zone 1 being the most proximal to the cell body and zone 10 being the most distal. HSV-VP26-RFP particles were counted in 6 adjacent microgrooves and averaged for each zone (36 axons for WT, 36 axons for Het and 30 axons for KI).

### Biostatistics

Experiments were performed in triplicates. To compare means, we used t-test after normality testing with Shapiro-Wilk test. For non-normal distribution, we used Mann Whitney U test. Multiple comparisons were performed with one-way ANOVA followed by Tukey’s post-hoc test. To compare categorical variables we used chi-squared test.

### Data availability

The datasets generated and/or analyzed during the current study are available from the corresponding author on reasonable request.

## Electronic supplementary material

Supplementary Materials
