# Supplementary material for: Mutant torsinA in the heterozygous DYT1 state compromises HSV propagation in infected neurons and fibroblasts
Source: Sci Rep. 2018 Feb 2;8:2324. doi: 10.1038/s41598-018-19865-2 (PMC5797141; doi:10.1038/s41598-018-19865-2)
Supplement: Supplementary file 1 — Supplementary Materials [file 41598_2018_19865_MOESM1_ESM.pdf]

Mutant torsinA in the heterozygous DYT1 state compromises HSV propagation in infected neurons and fibroblasts

Bence György[1], Lilian Cruz[1], David Yellen[1], Massimo Aufiero[1], Isabel Alland[1] Xuan Zhang[1], Maria Ericsson[2], Cornel Fraefel[3], Yu-Ching Li[4], Shuko Takeda[5], Bradley T. Hyman[1,5], Xandra O. Breakefield[1,*]

[1]Molecular Neurogenetics Unit, Department of Neurology and Center for Molecular Imaging Research, Department of Radiology, Massachusetts General Hospital and Program in Neuroscience, Harvard Medical School, Boston, MA 02114 USA; [2]Department of Cell Biology, Harvard Medical School, Boston, MA 02115 USA; [3]Institute of Virology, University of Zurich, Zurich, 8057, Switzerland; [4]Department of Neurology, College of Medicine, University of Florida, Gainesville, FL USA 32610; [5]Department of Neurology, Massachusetts General Hospital, Massachusetts General Institute of Neurodegenerative Diseases (MIND), Charlestown, MA 02129 USA and Department of Neurology, Harvard Medical School, Boston MA 02114 USA and Massachusetts Alzheimer Disease Research Center, Charlestown, MA 02129 USA

[*]Corresponding author: Xandra O. Breakefield, Ph.D., Molecular Neurogenetics Unit, Massachusetts General Hospital-East, 13th Street, Building 149, Charlestown, MA 02129 USA, Phone: 617-726-5728, Fax: 617-724-1537, Email: breakefield@hms.harvard.edu

Running title: Mutant torsinA interferes with Herpes simplex virus type 1 propagation

Supplementary Table 1.

Conditions		Total	Infected	Uninfected	Replic. centers	HSV in NE	Blebs*
Uninfected	WT	50	-	50	-	-	0[g]
	Het	50	-	50	-	-	1[g]
	KI	50	-	50	-	-	13[g]
8h	WT	30	21[a]	9	0[c]	4[e]	2[h]
	Het	30	27[a]	3	1[c]	2[e]	4[h]
	KI	30	29[a]	1	0[c]	12[e]	11[h]
15h	WT	30	28[b]	2	11[d]	2[f]	6[i]
	Het	30	27[b]	3	5[d]	4[f]	4[i]
	KI	30	27[b]	3	6[d]	6[f]	16[i]

*Number of cells with 3 blebs or more

Supplementary Table 1. Cells were counted based on electron microscopic images. To compare genotypes in the case of each variable, we applied chi-square test: a: p=0.009** (infected vs. non-infected), b: n.s. (infected vs. non-infected), c: n.s. (replication centers vs no replication centers), d: n.s. (replication centers vs. no replication centers), e: p=0.009** (HSV in NE vs no HSV in NE, only infected cells were analyzed), f: n.s. (HSV in NE vs no HSV in NE, only infected cells were analyzed), g: p<0.0001*** (blebs vs no blebs), h: p=0.008** (blebs vs no blebs), i: p=0.001* (blebs vs no blebs).

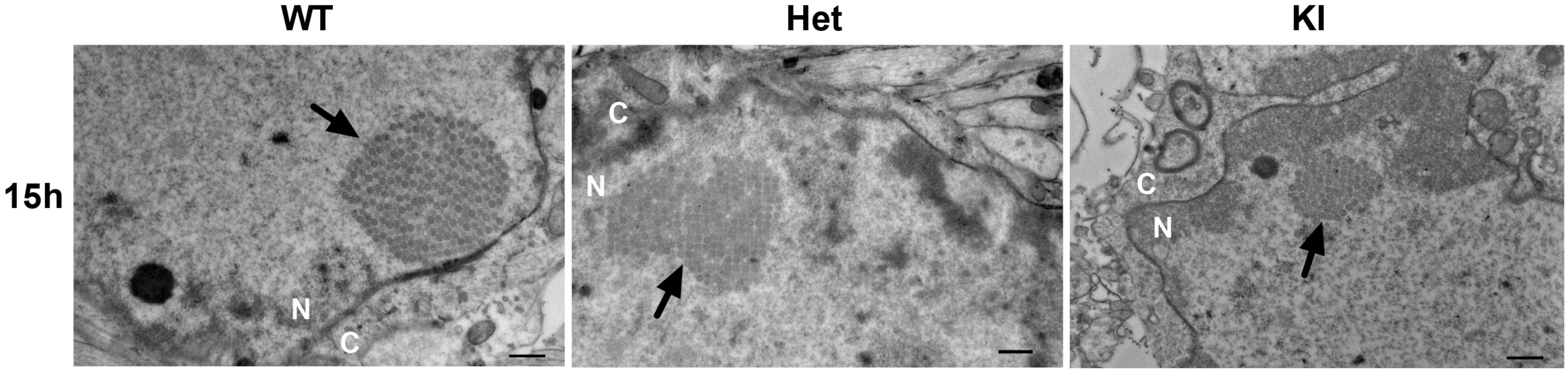

Supplementary Figure 1. HSV replication centers in the nuclei of DYT1 neurons. Wild-type (WT), heterozygous (Het), and homozygous KI for the DYT1 mutation (KI) neurons were infected with HSV (M.O.I. 1) and imaged using an electron microscope at 15 h post-infection. Black arrows indicate HSV replication centers in the nucleus. N: nucleus; C: cytoplasm. Scale bars: 500 nm.

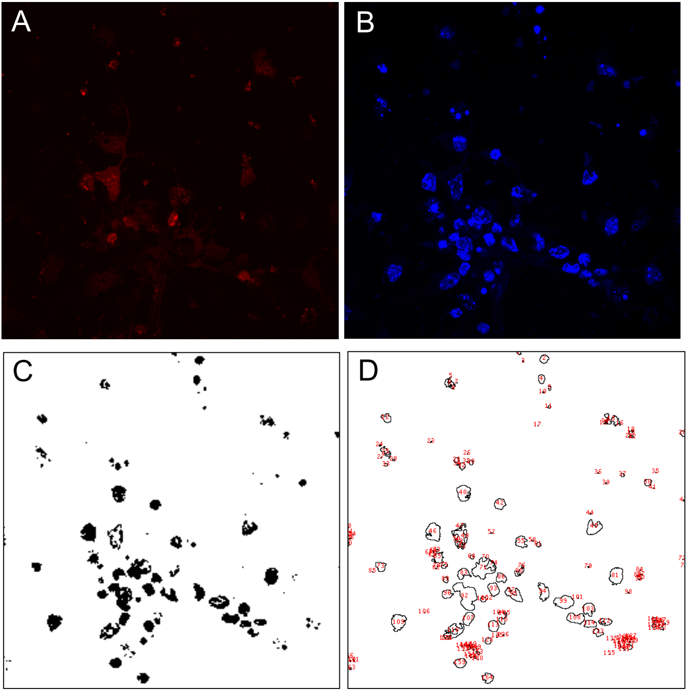

Supplementary Figure 2. Cytoplasmic intensities of HSV-VP26-RFP in WT, Het and KI neurons were analyzed by measuring the intensity of the red fluorescence signal in the entire area outside the nucleus using ImageJ v2.0.0 by the following steps: First, we measured the integrated density of the entire image in the red channel (A). Then, we visualized the image in the DAPI channel (B) and used the threshold tool to highlight the nuclei. Next, we converted the nuclei area into a grayscale binary image (C) and measured the red fluorescence intensity in the nuclei regions using the "Analyze Particles" tool. (D).Finally, the nuclear integrated density values were subtracted from the total fluorescence of the image to determine the mean cytoplasmic fluorescence intensity.

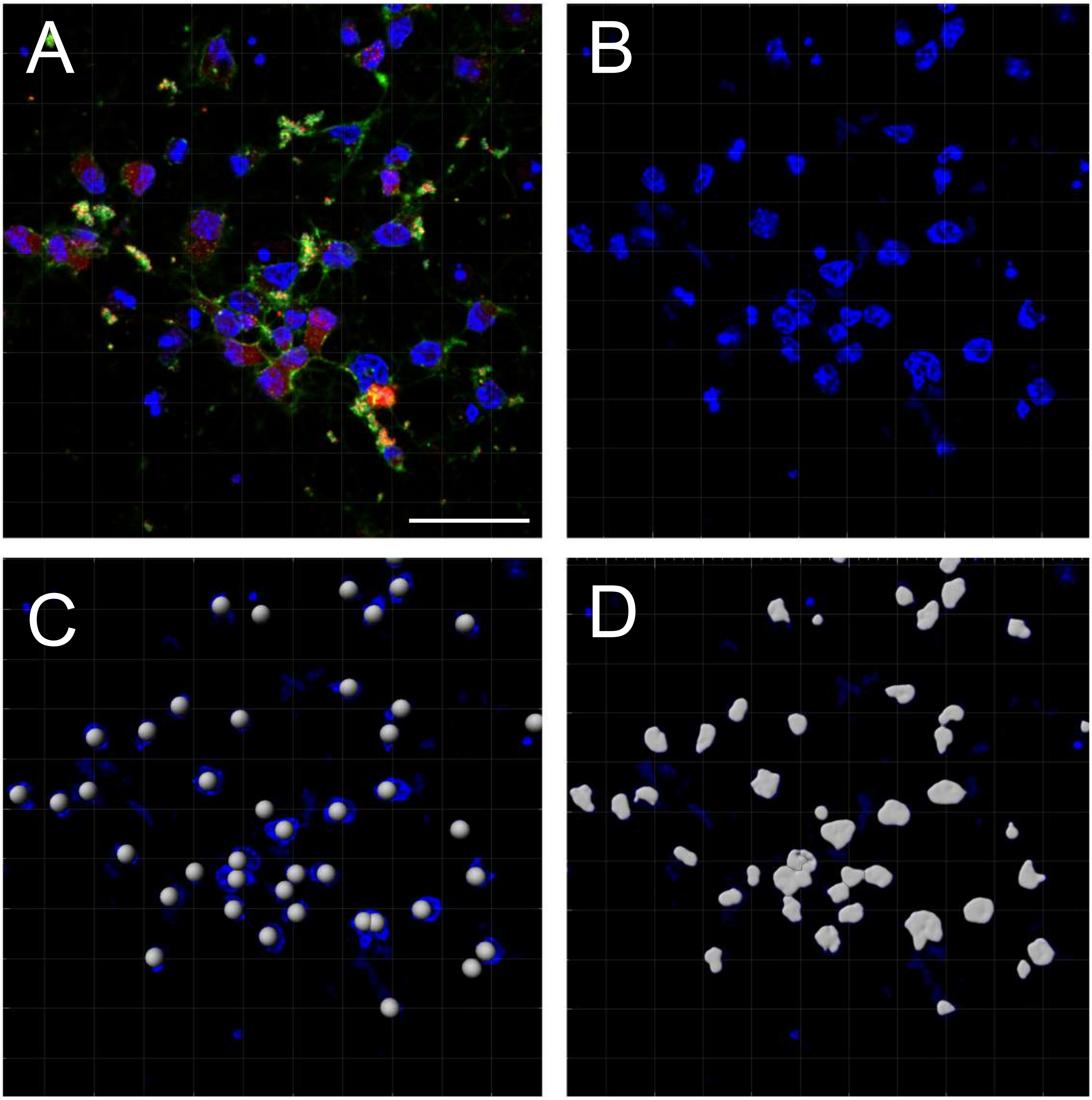

Supplementary Figure 3. Method for quantification of mean nuclear fluorescence intensity in HSV-VP26-mRFP infected neurons. Images were acquired using a Zeiss LSM 710 confocal microscope using a voxel size of 0.4393x0.4393x1 micron^3. Cells were stained for DAPI (blue), WGA488 (green) and virus is shown in red. Scale bar is 50 µm. Measurement was carried out with Imaris 9.0.0. software.(A) Raw images were loaded into the program. (B) Cell nuclei were visualized in the blue channel (DAPI staining). (C) Seed-points were defined for automatic single cell nuclear detection. (D) Nuclear area was assigned by the program and fluorescent intensity was determined for every cell separately. Averages of nuclear mean fluorescent intensities are shown on Figure 2B-D.
